# The effect of non-invasive brain stimulation combined with motor imagery on upper limb motor function and activities of daily living in stroke patients: a systematic review and meta-analysis

**DOI:** 10.3389/fneur.2026.1807447

**Published:** 2026-06-03

**Authors:** Juhao Hou, Yi Fang, Shilin Wen

**Affiliations:** Capital University of Physical Education and Sports, Beijing, China

**Keywords:** meta-analysis, motor imagery, non-invasive brain stimulation, repetitive transcranial magnetic stimulation, stroke, transcranial direct current stimulation

## Abstract

**Introduction:**

Severe stroke frequently leads to adult disability and a loss of functional independence. Specifically, stroke-induced upper limb motor impairment significantly compromises the quality of daily life for these patients. Therefore, evaluating the efficacy of non-invasive brain stimulation combined with motor imagery for the recovery of upper limb motor function is critical for improving patient outcomes.

**Methods:**

A systematic search for relevant randomized controlled trials (RCTs) was conducted across PubMed, Cochrane Library, ScienceDirect, Web of Science, EMBASE, CNKI, Wanfang Data, and VIP databases, spanning from their inception through September 4, 2025. Key outcome measures included upper extremity motor function (assessed via FMA-UE), upper extremity functional activity (ARAT and WMFT), and activities of daily living (MBI). Statistical analyses were performed using Stata 18.0. Effect sizes were calculated as weighted mean differences (WMD) or standardized mean deviations (SMD) with 95% confidence intervals (CI) using random-effects models.

**Results:**

The meta-analysis, comprising 21 studies from 16 articles, demonstrated that MI combined with NIBS significantly improved upper extremity motor function (WMD = 5.75; 95% CI = 3.67, 7.82; *P* < 0.001), upper extremity functional activity (SMD = 1.18; 95% CI = 0.78, 1.57; *P* < 0.001), and activities of daily living (WMD = 8.94; 95% CI = 4.36, 13.53; *P* < 0.001). Subgroup analyses identified stroke chronicity, NIBS modality, stimulation and training duration, MI paradigm, and comparator type as significant moderators of the intervention effect.

**Conclusion:**

The combined intervention of MI and NIBS constitutes an effective and safe rehabilitation strategy that significantly enhances upper limb motor function and activities of daily living following stroke. Nevertheless, further high-quality, large-scale randomized controlled trials are warranted to validate these findings and establish standardized clinical protocols.

**Systematic review registration:**

PROSPERO (Registration number: CRD420251167952).

## 1 Introduction

Stroke remains the leading cause of long-term disability among adults globally (1). Characterized by high morbidity, this acute cerebrovascular disease often precipitates severe motor dysfunction, such as hemiplegia and muscle weakness, which severely compromises patients' functional independence (2). Consequently, the condition imposes substantial economic pressure, physical strain, and psychological burden on individuals and their families (3). Furthermore, from a socio-economic perspective, stroke poses a severe public health challenge its high incidence and disability rates have led to staggering direct medical expenses and indirect productivity losses (4). In particular, upper limb motor dysfunction—especially the loss of fine motor skills—constitutes a critical determinant of patients' independence, quality of life, and societal reintegration (5).

The efficacy of traditional rehabilitation for stroke patients with severe functional impairment is often constrained by challenges such as the inability to execute therapeutic movements, premature therapeutic plateaus, and insufficient generalization of training gains to daily life (6). To address these challenges, central intervention strategies, particularly motor imagery (MI), have received increasing attention. MI involves the mental rehearsal of motor tasks without overt muscle contraction; this process activates brain networks overlapping with those of actual movements and is regarded as effective for promoting neural plasticity. However, studies indicate significant individual variability in the efficacy of MI, which is contingent upon the vividness and stability of the patient's imagery ability. Consequently, for patients with cognitive impairment or deficits in imagery generation, the therapeutic benefit is often limited (7). Conversely, non-invasive brain stimulation (NIBS) offers a novel rehabilitative approach by modulating cortical excitability and restoring interhemispheric inhibitory balance post-stroke. In the context of this study, NIBS is operationally defined to specifically encompass transcranial direct current stimulation (tDCS) and repetitive transcranial magnetic stimulation (rTMS). To address the post-stroke interhemispheric imbalance, different NIBS modalities prime the motor network through distinct mechanisms: unilateral anodal tDCS (a-tDCS) directly upregulates ipsilesional excitability; low-frequency rTMS (LF-rTMS) downregulates contralesional hyperexcitability to relieve transcallosal inhibition; and bilateral tDCS (bi-tDCS) concurrently applies both effects. Yet, evidence indicates that without concurrent targeted functional training, NIBS-induced physiological changes often fail to translate into sustained functional gains (8). In recent years, the synergistic integration of NIBS and MI has attracted significant attention. Mechanistically, NIBS can lower the activation threshold of the ipsilesional motor cortex, thereby priming the target motor network for more robust activation during subsequent MI. Preliminary clinical studies suggest that this combined strategy demonstrates superior efficacy compared to monotherapy in improving upper limb motor function, motor control, and activities of daily living (9, 10).

Research indicates that in stroke rehabilitation, MI-induced improvement in upper limb function is phase-dependent, being more pronounced in the early and chronic phases but less significant in the late subacute phase. Furthermore, MI demonstrates greater efficacy in patients with moderate-to-severe functional deficits, whereas it yields limited benefit in those with mild deficits (11). One study investigating MI priming found that initiating treatment with MI produces a marginal benefit for upper limb mobility; conversely, movement observation combined with MI produced negligible improvement (12). NIBS research in stroke rehabilitation has evolved from purely mechanistic explorations to inclusion in clinical guideline recommendations (13). rTMS has been shown to play a significant role in facilitating upper extremity motor recovery after stroke (14). While NIBS improved upper extremity Fugl-Meyer Assessment (FMA) scores, the Barthel Index for activities of daily living (ADL) remained unchanged, implying that isolated brain stimulation may be insufficient to translate motor gains into complex functional enhancements (15). Notably, some evidence suggests that NIBS combined with MI does not confer a significant additive benefit compared to stimulation alone (16). Given the paucity of reviews addressing NIBS combined with MI, and the lingering controversy regarding the comparative efficacy of monotherapies vs. combined interventions, it is crucial to conduct an updated meta-analysis to integrate recent evidence. The rationale for combining NIBS with MI centers on correcting interhemispheric imbalance and driving Hebbian plasticity. Distinct NIBS modalities prime the motor network by modulating cortical excitability: a-tDCS upregulates the ipsilesional primary motor cortex (M1); LF-rTMS downregulates contralesional hyperexcitability to relieve transcallosal inhibition; and bi-tDCS concurrently applies both effects. However, NIBS-induced excitability lacks task-specificity. MI provides the necessary synchronous, endogenous activation of targeted motor networks. Guided by Hebbian plasticity rules, pairing the exogenous postsynaptic priming of NIBS with the task-specific presynaptic input of MI facilitates robust long-term potentiation (LTP)-like plasticity. This temporal synergy optimizes sensorimotor integration, effectively translating transient cortical modulation into sustained functional motor recovery.

Consequently, the primary objective of this study is to systematically evaluate the efficacy of non-invasive brain stimulation (NIBS) combined with motor imagery (MI) for the recovery of motor function in stroke patients. We proposed two research hypotheses: (1) NIBS combined with MI will yield superior therapeutic outcomes compared to either intervention administered as a monotherapy. (2) The magnitude of the therapeutic effect will be significantly modulated by intervention parameters, such as treatment protocols and dosage, as identified through subgroup analysis.

## 2 Methods

The protocol for this systematic review was prospectively registered on PROSPERO (registration number: CRD420251167952) and the study was conducted in accordance with the Preferred Reporting Items for Systematic Reviews and Meta-Analyses (PRISMA) guidelines (17).

### 2.1 Date sources and search strategy

Two independent investigators systematically searched PubMed, Cochrane Library, ScienceDirect, Web of Science, EMBASE, CNKI, Wanfang Data, and VIP databases from their inception through September 4, 2025. Additionally, the reference lists of all included articles were manually screened to identify eligible studies that may not have been captured by the database search. No restrictions were imposed regarding publication country or language. The search strategy utilized a combination of the following Medical Subject Headings (MeSH) and free-text terms: “transcranial magnetic stimulation”, “transcranial direct current stimulation” (tDCS), “repetitive transcranial magnetic stimulation” (rTMS), “non-invasive brain stimulation”, “motor imagery” (MI), “apoplexy”, and “stroke”. Detailed in Table 1.

**Table 1:** Basic information of the literature.

Author (Reference)	Country	Type of stroke (I/H)	Age (mean ±SD)	Affect limb (L/R)	Duration of disease (mean ±SD)	Intervention	Intervention duration (number of sessions)	Outcomes
Hong et al. (16)	Singapore	NR	EG: 56.64 ± 9.59	EG:4/5	EG:33.9 ± 24.6	EG:tDCS+MI	4weeks	DTI CBF FMA RMT
			CG_1_: 56.64 ± 9.59	CG_1_:3/6	CG_1_: 33.3 ± 15.1	CG_1_:MI		
Kashoo et al. (36)	Saudi Arabia	EG: 4/28	EG: 58.7 ± 5.7	EG:14/18	EG: 7.8 ± 1.3	EG: tDCS+MI	2weeks	Fugl-Meyer ARAT
		CG_1_: 2/30	CG_1_: 59.9 ± 5.6	CG_1_:15/12	CG_1_: 7.4 ± 1.2	CG_1_: Sham stimulation+MI		
Yu et al. (42)	China	EG: 2/12	EG: 54.93 ± 8.73	EG:4/10	EG: 38.00 ± 26.76	EG: KI-BCI/tDCS	5 d/w, 4 weeks	FMAMSS MBI ARAT
		CG_1_: 5/10	CG_1_: 56.13 ± 8.63	CG_1_:4/11	CG_1_: 44.07 ± 22.63	CG_1_: tDCS		
		CG_2_: 4/10	CG_2_: 60.79 ± 11.48	CG_2_:5/9	CG_2_: 39.36 ± 12.67	CG_2_: KI-BCI		
Pan et al. (37)	China	NR	EG: 63.38 ± 6.45	NR	EG: 4.96 ± 1.07	EG: LF-rTMS+MI	5 d/w, 2 weeks	WMFT FMA MBI BBT
			CG_1_: 64.14 ± 4.49		CG_1_: 5.13 ± 1.09	CG_1_: LF-rTMS		
Gao et al. (38)	China	EG: 22/23	EG: 55.8 ± 10.9	EG:24/21	EG: 33.6 ± 12.9	EG: tDCS+MI	6 d/w, 4 weeks	FMA FTHUE-HK sEMG
		CG_1_: 23/22	CG_1_: 56.1 ± 10.7	CG_1_:25/20	CG_1_: 33.9 ± 13.3	CG_1_: tDCS		
Chew et al. (39)	Singapore	EG: 4/6	EG: 52.2 ± 11.8	EG:5/5	EG: 31.3 ± 24.5	EG: tDCS+MI-BCI	2 weeks	UE-FM RMT
		CG_1_: 2/7	CG_1_: 56.4 ± 9.6	CG_1_:3/6	CG_1_: 33.3 ± 15.1	CG_1_: Sham stimulation+MI-BCI		
Xingwang et al. (43)	China	EG: 11/30	EG: 61.49 ± 10.50	NR	NR	EG: tDCS+MI	5 d/w, 4 weeks	FMAMBI
		CG_1_:12/27	CG_1_: 58.77 ± 14.30			CG_1_: tDCS		
Liang et al. (49)	China	EG: 6/19	EG: 56.36 ± 11.64	EG:7/18	EG: 60.12 ± 17.88	EG: rTMS+MI	5 d/w, 4 weeks	MEP CMCT FMA ARAT MBI
		CG_1_: 8/17	CG_1_: 54.68 ± 12.03	CG_1_:5/15	CG_1_: 56.28 ± 13.53	CG_1_: rTMS		
		CG_2_: 5/20	CG_2_: 51.28 ± 11.61	CG_2_:12/13	CG_2_: 57.40 ± 10.83	CG_2_: MI		
Chunzhen et al. (44)	China	NR	EG: 57.9 ± 9.8	NR	EG: 8.0 ± 1.9	EG: tDCS+MI	3 weeks	FMAMBI PSQI
			CG_1_: 57.6 ± 10.2		CG_1_: 8.4 ± 2.2	CG_1_: tDCS		
Shasha et al. (50)	China	EG: 8/20	EG: 66.18 ± 3.98	EG:0/28	EG: 62.86 ± 12.67	EG: tDCS+MI	6 d/w, 4 weeks	FMAWMFT MBI MoCA
		CG_1_: 10/18	CG_1_: 65.86 ± 3.97	CG_1_:0/28	CG_1_: 61.21 ± 14.36	CG_1_: MI		
		CG_2_: 13/15	CG_2_: 66.21 ± 4.06	CG_2_:0/28	CG_2_: 56.43 ± 10.18	CG_2_: tDCS		
Yulin et al. (48)	China	EG: 27/25	EG: 63.22 ± 6.41	EG:28/24	EG: 6.31 ± 1.36	EG: tDCS+MI	7 d/w, 4 weeks	NIHSSLOTCA FMA MBI
		CG_1_: 29/23	CG_1_: 62.75 ± 7.04	CG_1_:27/25	CG_1_: 7.04 ± 1.46	CG_1_: tDCS		
Tingting et al. (46)	China	EG: 8/26	EG: 53.08 ± 9.30	EG:21/13	EG: 13.19 ± 4.35	EG: rTMS+MI	6 d/w, 4 weeks	NIHSSMEP MEP-CL CMCT FMA MAS MBI
		CG_1_: 8/25	CG_1_: 51.98 ± 8.65	CG_1_:18/15	CG_1_: 13.28 ± 3.70	CG_1_: MI		
Zhou et al. (40)	China	EG: 15/17	EG: 54.25 ± 8.23	EG:16/16	EG: 36.16 ± 19.90	EG: tDCS+MI	6 d/w, 8 weeks	FMA FTHUE MBI
		CG_1_: 13/18	CG_1_: 52.29 ± 10.72	CG_1_:14/17	CG_1_: 36.47 ± 20.58	CG_1_: MI		
Leilei et al. (47)	China	EG: 13/17	EG: 46.1 ± 6.8	NR	EG: 14.6 ± 3.5	EG: rTMS+MI	5 d/w, 4 weeks	FMA FTHUE-HK CL CMCT
		CG_1_: 16/14	CG_1_: 44.3 ± 6.6		CG_1_: 14.0 ± 3.9	CG_1_: MI		
Jia et al. (41)	China	EG: 3/16	EG: 58.42 ± 8.82	EG:10/9	EG: 16	EG: rTMS+GMI	5 d/w, 4 weeks	FMA ARAT MBI MAL-AOU MAL-QOM MEP
		CG_1_: 3/16	CG_1_: 61.89 ± 9.43	CG_1_:9/10	CG_1_: 17	CG_1_: GMI		
		CG_2_: 3/15	CG_2_: 55.56 ± 11.28	CG_2_:7/11	CG_2_: 21	CG_2_: rTMS		
Feifei et al. (45)	China	NR	EG: 58.84 ± 7.37	EG:24/17	EG: 27.28 ± 4.36	EG: rTMS+GMI	7 d/w, 4 weeks	FMA TUGT 6MWT PASS MBI NIHSS ADC
			CG_1_: 57.36 ± 7.85	CG_1_:23/18	CG_1_: 28.31 ± 5.32	CG_1_: GMI		
			CG_2_: 56.32 ± 7.98	CG_2_:25/16	CG_2_: 27.04 ± 5.79	CG_2_: rTMS		

I, infarction; H, hemorrhage; L, left; R, right; SD, standard deviation; EG, experimental group; CG, control group; MI, motor imagery; rTMS, repetitive transcranial magnetic stimulation; tDCS, transcranial direct current stimulation; FMA, Fugl-Meyer assessment; FMA-UE, Fugl-Meyer assessment of upper extremity; FTHUE, functional test for the hemiplegic upper extremity; MBI, modified Barthel index; RMT, resting motor threshold; NIHSS, national institute of health stroke scale; MEP-CL, cortical latency of motor evoked potential; CMCT, central motor conduction time; TUGT, time up and go test; MAS, motor assessment scale; WMFT, wolf motor function test; MAL-AOU, motor activity log-amount of use; MAL-QOM, motor activity log-quality of movement; MMSE, minimum mental state examination; MoCA, montreal cognitive assessment; LOTCA, Loewenstein occupational therapy cognitive assessment; FTHUE-HK, Hong Kong version of the hemiplegic upper limb functional test; m, months; s, simultaneously; d, day; BBT, box and blocks test; ARAT, action research arm test; DTI, diffusion tensor imaging; CBF, cerebral blood flow; MEP, motor evoked potential; w, weeks; d, days.

### 2.2 Inclusion and exclusion criteria

Studies were included if they met the following criteria: (1) Population: adult stroke survivors exhibiting upper limb motor impairment; (2) Intervention: protocols utilizing tDCS or rTMS combined with MI. Recognizing the clinical variability in MI delivery, eligible MI protocols were systematically categorized into three distinct paradigms for subgroup analysis: General-MI (conventional guided imagery), BCI-MI (Brain-Computer Interface-assisted), and GMI (Graded Motor Imagery); (3) Comparator: control groups receiving monotherapy (tDCS, rTMS, or MI alone); (4) Outcome measures: quantitative assessments including the Fugl-Meyer Assessment-Upper Extremity (FMA-UE), Modified Barthel Index (MBI), Wolf Motor Function Test (WMFT), or Action Research Arm Test (ARAT); and (5) Study design: only randomized controlled trials (RCTs) were considered for inclusion. Conversely, studies were excluded based on the following criteria: (1) Participants did not exhibit upper limb motor deficits; (2) The intervention utilized stimulation modalities other than NIBS in combination with MI; (3) The publication type was a review, conference abstract, or case report; (4) Relevant outcome data were not reported or were incompatible with the selected measures; or (5) Insufficient data were available for extraction.

### 2.3 Data extraction

Data were independently extracted by two investigators using a standardized form and included: study characteristics (first author, publication year, country, and design); participant demographics (age, stroke etiology, affected limb, and chronicity); intervention protocols (NIBS modality, parameters [frequency, intensity, session duration], MI paradigm, and dosage); the temporal relationship between MI and NIBS; comparator group type; total intervention duration; and reported outcomes, including safety data (adverse events). We standardized the chronicity classification across all extracted data based on the consensus guidelines from the Stroke Recovery and Rehabilitation Roundtable (SRRR), categorizing patients into acute/subacute ( ≤ 6 months) and chronic (>6 months) phases.

### 2.4 Quality assessment

The risk of bias for included studies was evaluated using the Cochrane Collaboration's Risk of Bias tool (18). The following domains were assessed: random sequence generation, allocation concealment, blinding of participants and personnel, blinding of outcome assessment, incomplete outcome data, selective reporting, and other sources of bias. Any discrepancies between the two primary reviewers were resolved through consultation with a third investigator. Analyses were performed using Review Manager software. Risk of bias was categorized as “low risk” (green), “high risk” (red), or “unclear risk” (yellow).

### 2.5 Statistical analysis

Quantitative data synthesis was performed using Stata 18.0, with 95% confidence intervals (CI) calculated for all outcomes. Statistical heterogeneity was evaluated using the Chi-square test (significance level *P* < 0.05) and quantified via the *I*^2^ statistic, where values of 25%, 50%, and 75% indicated low, moderate, and high heterogeneity, respectively (19). Continuous outcomes were pooled using Weighted Mean Differences (WMD) or Standardized Mean Differences (SMD). A fixed-effects model was applied when heterogeneity was low (*I*^2^ < 50%), whereas a random-effects model was employed for studies exhibiting significant heterogeneity. Continuous outcomes were pooled based on the uniformity of the assessment instruments. Weighted Mean Differences (WMD) were utilized when outcomes were evaluated using identical scales across studies. Conversely, Standardized Mean Differences (SMD) were employed when different measurement tools were used to assess the same underlying construct. To investigate sources of heterogeneity, subgroup analyses were conducted based on: (1) comparator type; (2) stroke chronicity; (3) NIBS modality; (4) NIBS duration; (5) MI paradigm; and (6) MI dosage. Additionally, sensitivity analyses were performed to ensure the robustness of results, and funnel plots were inspected to assess potential publication bias.

## 3 Result

### 3.1 Literature screening

**Figure 1:**
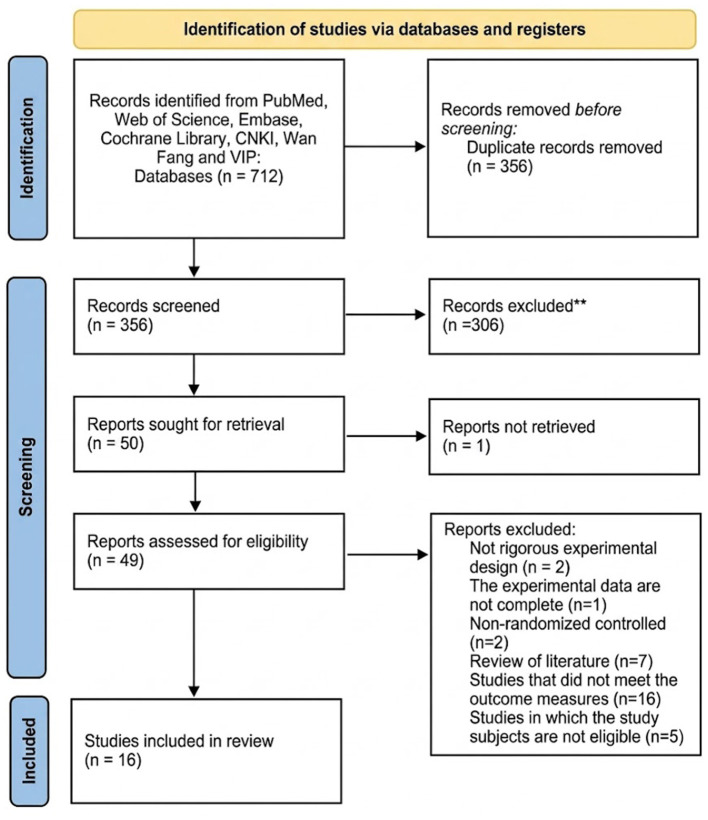
Flowchart of literature screening.

The initial search identified 712 records. After removing 365 duplicates, the remaining records were screened by title and abstract, resulting in the exclusion of 306 papers. Subsequently, 50 articles were excluded following full-text review. One additional study was excluded due to missing data, following unsuccessful attempts to contact the authors. Ultimately, 16 articles comprising 21 independent comparisons were included in the quantitative synthesis (Figure 1).

### 3.2 Basic information of the literature

Participant characteristics from the included trials are summarized in Table 1. The meta-analysis comprised 16 articles yielding 21 independent comparisons, representing a total cohort of 1,249 stroke survivors. Detailed intervention protocols are presented in Table 2. Regarding stroke etiology, four studies did not specify the subtype. The remaining studies included 526 patients with ischemic stroke and 335 with hemorrhagic stroke. Five studies did not report the affected hemisphere; however, right-sided hemiparesis was inferred for one study based on subject inclusion criteria. Across the reported cohorts, 288 patients exhibited left-sided hemiparesis and 390 exhibited right-sided hemiparesis. Notably, six articles employed a multi-arm design utilizing two distinct control groups. Control conditions varied: 11 comparisons utilized MI monotherapy, while 10 utilized NIBS monotherapy. Regarding motor imagery paradigms, 11 studies used conventional MI, 5 used Brain-Computer Interface-assisted MI (BCI-MI), and 4 used Graded Motor Imagery (GMI). Intervention duration ranged from 2 to 4 weeks for the majority of trials, with a single study extending to 8 weeks.

**Table 2:** Detailed interventions reported in the meta-analysis.

Study	Classification	NIBS parameters	MI parameters	Type of control group	Adverse events
Hong et al. (16)	Chronic (>9 months)	35 cm^2^, 1.0 mA × 20-min tDCS Anode: ipsilesional M1 Cathode: contralateral M1	BCI-MI 40 min	MI	None
Kashoo et al. (36)	Chronic (>6 months)	35 cm^2^, 1.5 mA × 30-min tDCS Anode: affected hemisphere C3/C4 Cathode: contralateral supraorbital	Visual assisted MI 30 min	MI	Tingling sensation, Itching under electrodes
Yu et al. (42)	Chronic (>6 months)	25 cm^2^, 2 mA × 20-min tDCS Anode: ipsilesional C3/C4 Cathode: contralateral C3/C4	Visual assisted MI/BCI-MI 30 min	KI-BCI/bi-tDCS	None
Pan et al. (37)	Chronic (3–12 months)	1 Hz over the M1 of the contralesional hemisphere, 90% RMT 1, 500 pulses	Audio-based MI 30 min	LF-rTMS	None
Gao et al. (38)	Chronic (>3 months)	35 cm^2^, 2 mA × 20-min tDCS Anode: ipsilesional C3/C4 Cathode: contralateral C3/C4	BCI-MI 40 min	bi-tDCS	None
Chew et al. (39)	Chronic, (>9 months)	35 cm^2^, 1 mA × 20-min tDCS Anode: ipsilesional M1 Cathode: contralateral M1	BCI-MI 20 min	Sham stimulation + MI-BCI	None
Xingwang et al. (43)	Acute (< 2 weeks)	1.5 mA × 20-min tDCS X2 (After 20 min, the cathode and anode switch positions) Anode: affected shoulder Cathode: contralateral M1	Verbal guidance,40 min	a-tDCS	None
Liang et al. (49)	Uncertain (1–6 months)	1 Hz over the M1 of the contralesional hemisphere, 90% RMT, 1,200 pulses	GMI,40 min	Sham stimulation-rTMS/GMI	None
Chunzhen et al. (44)	Uncertain	1.5 mA × 20-min tDCS X2 (After 20 min, the cathode and anode switch positions) Anode: ipsilesional M1 Cathode: contralateral M1	MI	bi-tDCS	None
Shasha et al. (50)	Acute (1–3 months)	35 cm^2^, 2 mA × 20-min tDCS Anode:DLPFC, F3 Cathode: orbitofrontal area, F8	Verbal guidance, 30 min	bi-tDCS/MI	None
Yulin et al. (48)	Acute (0.5–12 weeks)	1.5 mA × 30-min tDCS Anode: ipsilesional M1 Cathode: contralateral M1	Verbal guidance, 20 min X2/d	bi-tDCS	None
Tingting et al. (46)	Acute (< 4 weeks)	1 Hz for 20 pulses/sequence, 30 sequences/time, pause 5-s, 100% RMT, 10 min	Verbal guidance 30 min	MI	None
Zhou et al. (40)	Acute (1–3 months)	35 cm^2^, 2 mA × 20-min tDCS Anode: ipsilesional M1 Cathode: contralateral shoulder	Verbal guidance MI 40 min	MI	None
Leilei et al. (47)	Acute	1 Hz over the M1 of the contralesional hemisphere, 90% RMT, 1,200 pulses	Verbal guidance, 20 min	MI	None
Jia et al. (41)	Uncertain (< 6 months)	1 Hz over the M1 of the contralesional hemisphere, 80% RMT, 20 min	GMI 30 min	rTMS/GMI	None
Feifei et al (45)	Acute	1 Hz over the M1 of the contralesional hemisphere, 90% RMT, 1,200 pulses	Verbal guidance, 20 min	rTMS/GMI	Headache negative emotions muscle aches epileptic seizures

### 3.3 Risk analysis of bias included in the study

**Figure 2:**
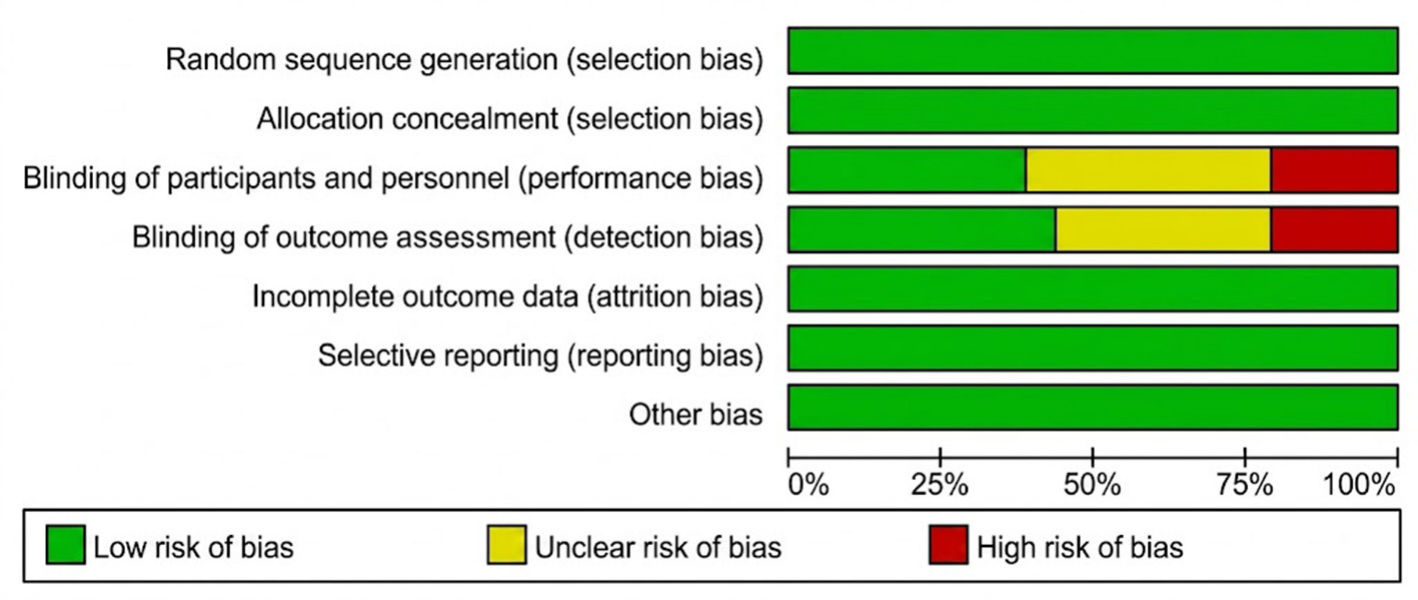
Risk bias diagram.

**Figure 3:**
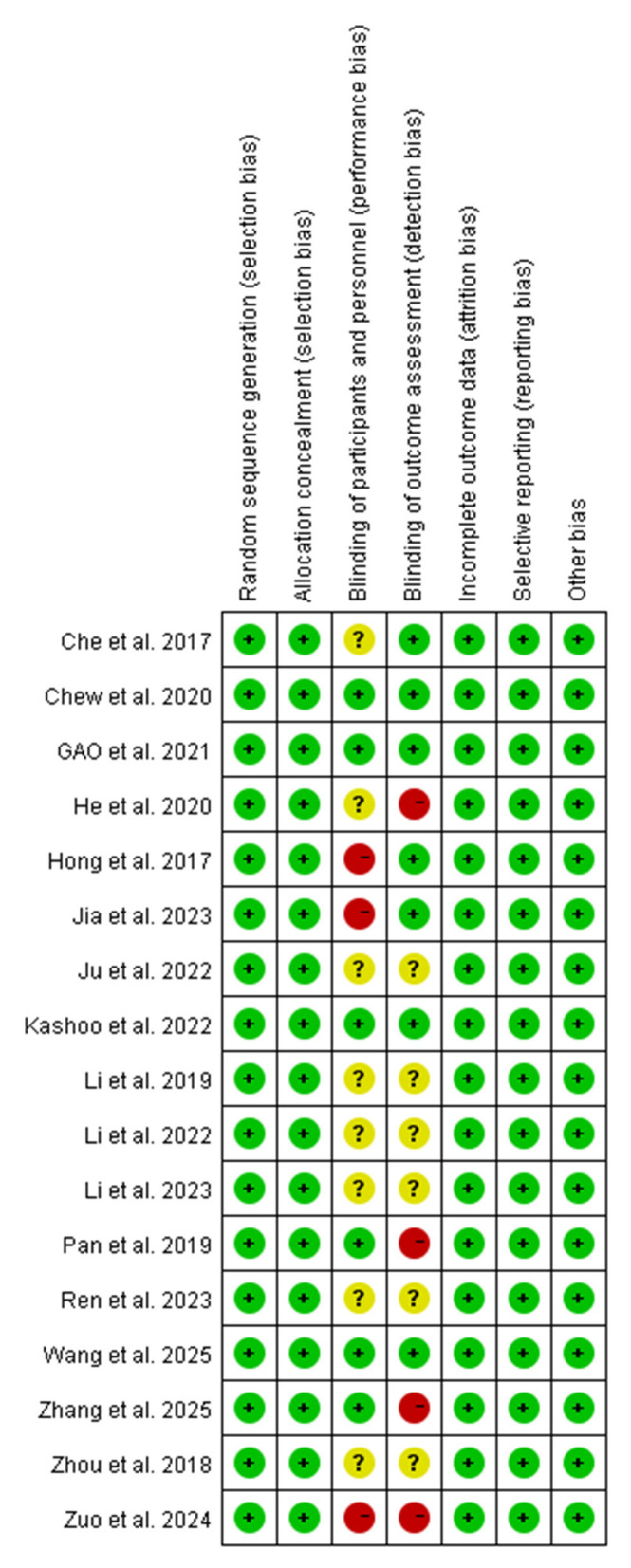
Summary of bias risk.

Figure 2 illustrates the risk of bias assessment across the 16 included studies. All studies utilized a randomized controlled design, and allocation concealment was reported in all 16 cases. Blinding of participants and personnel was confirmed in six studies, whereas the remaining studies did not explicitly report this status. Blinded outcome assessment was documented in seven studies. Complete outcome data were reported for all studies. Figure 3 presents the summary of risk of bias.

### 3.4 Meta-analysis

#### 3.4.1 Upper limb motor function

**Figure 4:**
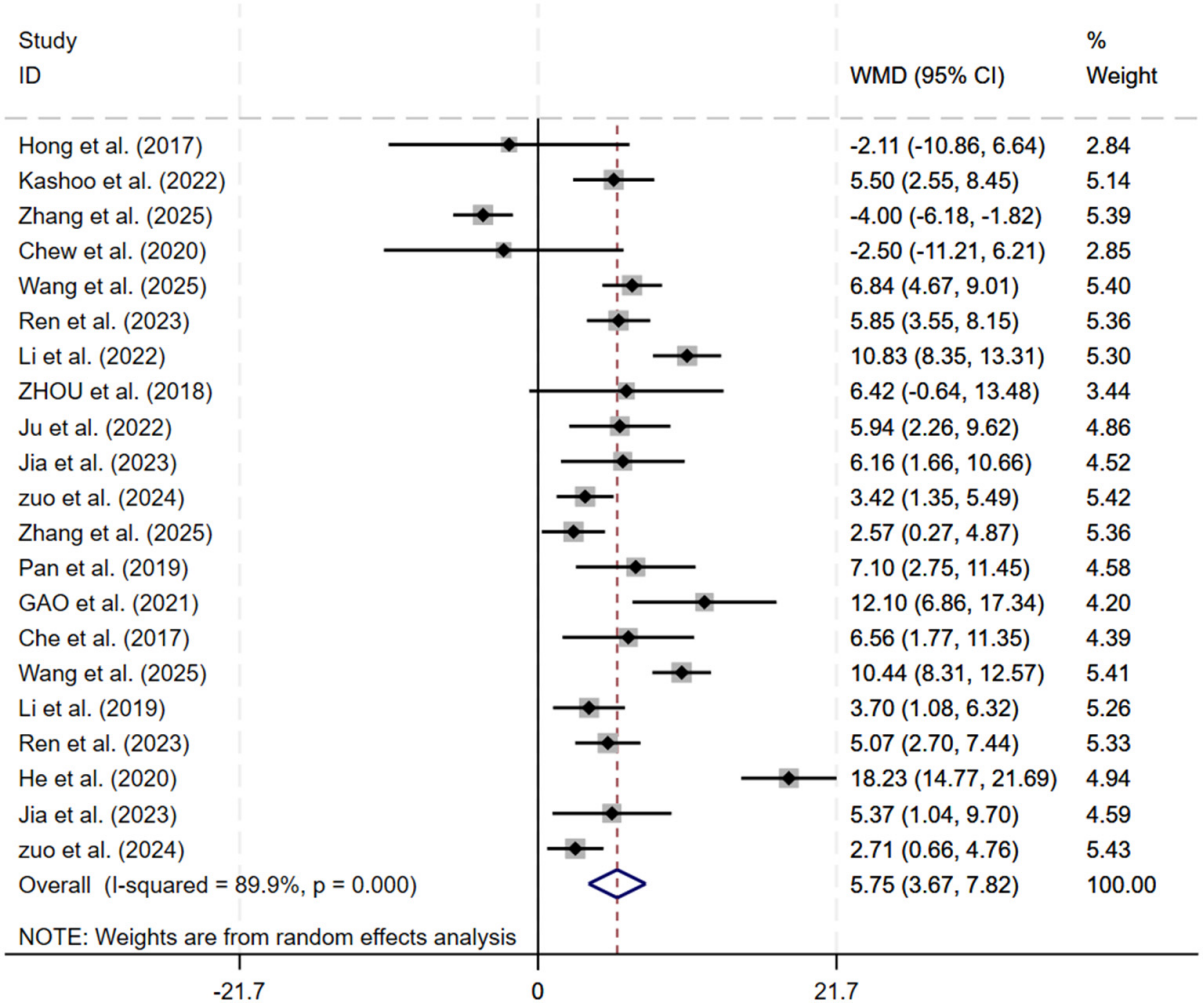
Forest plot of motor function of the upper limbs.

Twenty-one studies utilized the FMA-UE as the primary outcome measure to assess upper limb motor function recovery in stroke patients. Significant heterogeneity was observed across the included studies (*I*^2^ = 89.9%, *P* < 0.001); consequently, a random effects model was adopted for analysis. The improvement in motor function in the MI combined with NIBS group was significantly greater than that in the control group (WMD = 5.75; 95% CI 3.67, 7.82; *P* < 0.001). These results are detailed in Figure 4.

#### 3.4.2 Functional activities of upper limb movement

**Figure 5:**
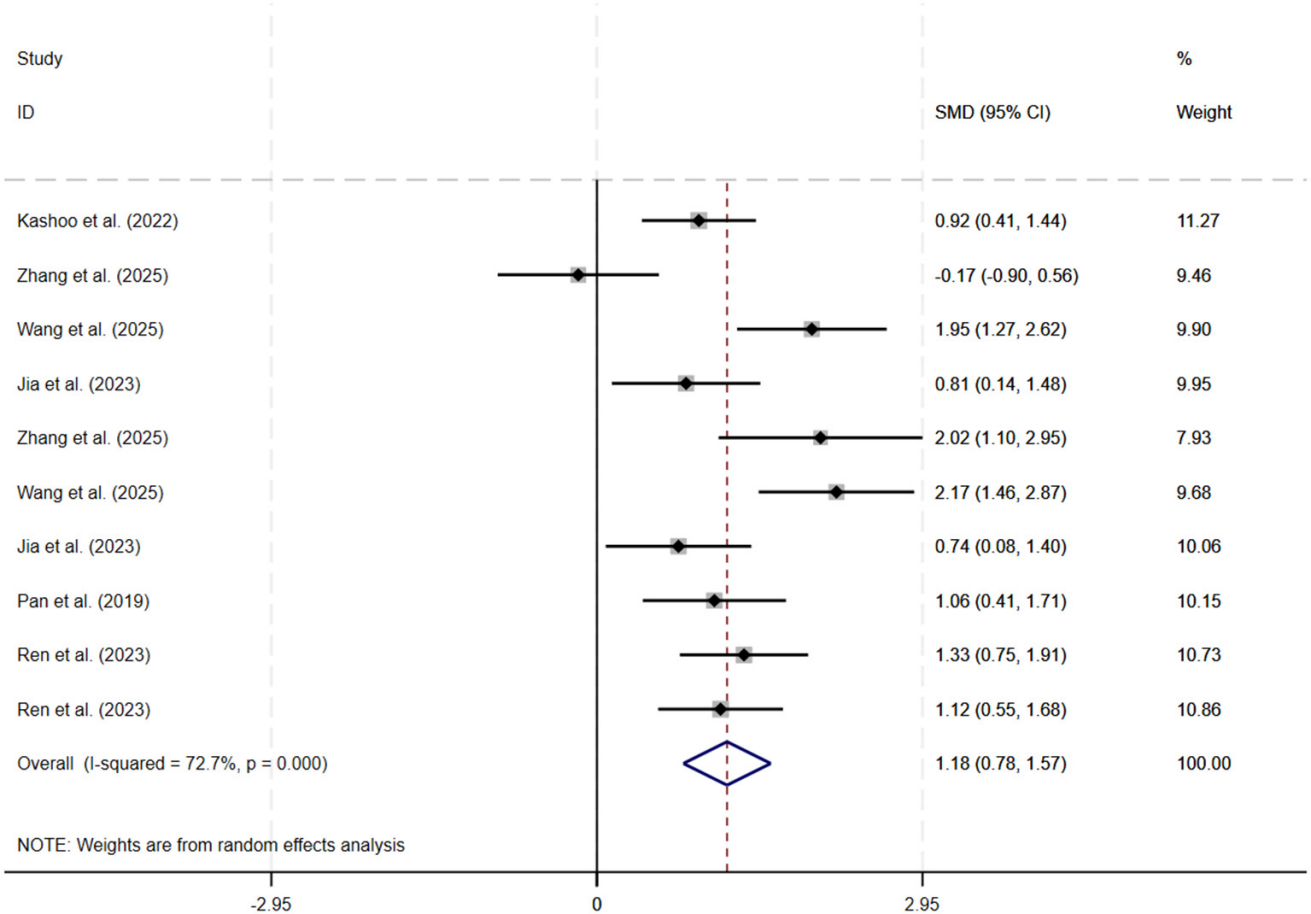
Forest plot of functional activities of upper limb movement.

Ten studies evaluated upper limb functional capacity: three utilized the Wolf Motor Function Test (WMFT) and seven employed the Action Research Arm Test (ARAT). Significant heterogeneity was detected among the included studies (*I*^2^ = 72.7%, *P* < 0.001); therefore, a random effects model was used for analysis. Overall, upper limb functional capacity improved significantly in the MI combined with NIBS group compared to the control group (SMD = 1.18; 95% CI 0.78, 1.57; *P* < 0.001), as illustrated in Figure 5.

#### 3.4.3 Activities of daily life

**Figure 6:**
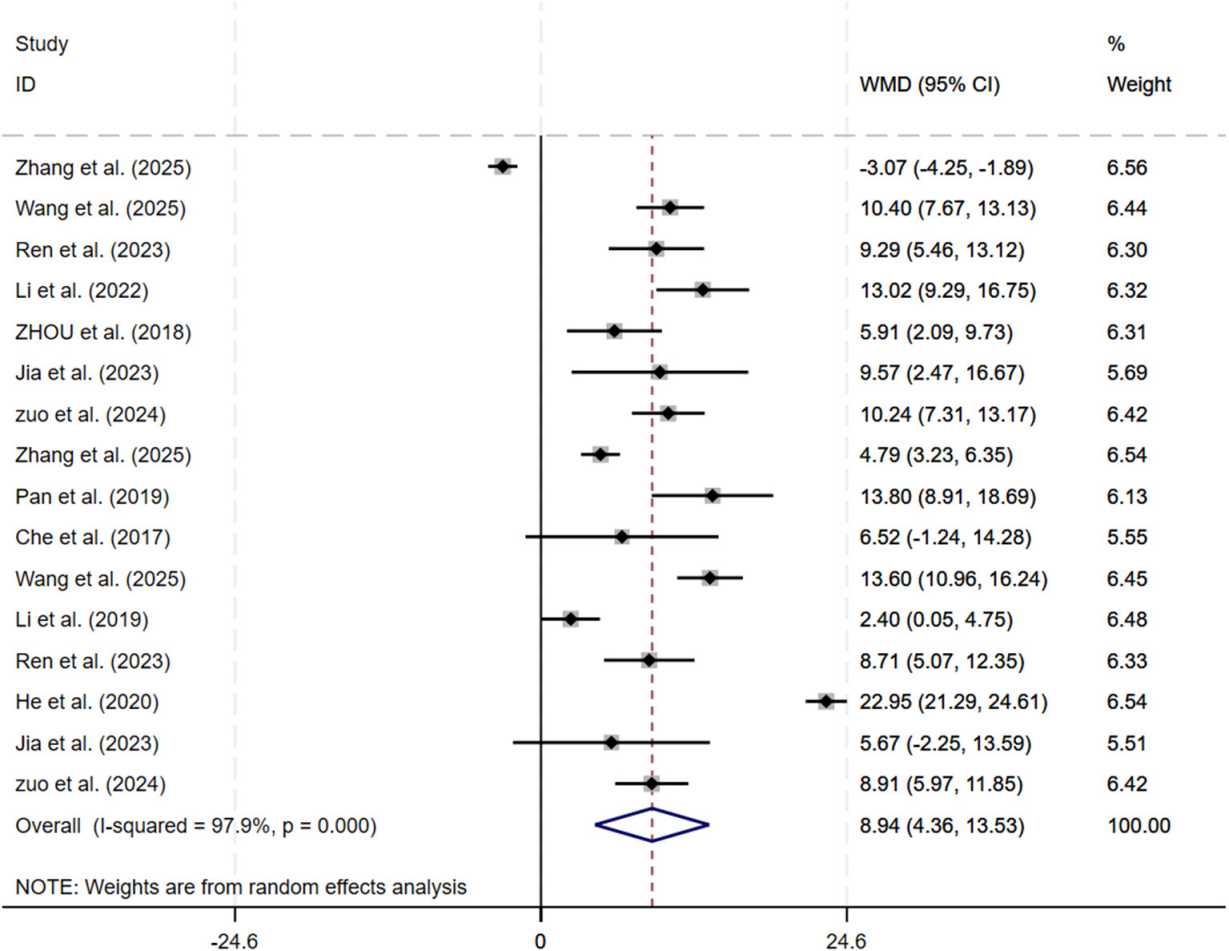
Forest plot of activities of daily living.

Nineteen studies utilized the Modified Barthel Index (MBI) to assess activities of daily living (ADL) in stroke patients. Substantial heterogeneity was observed across the included studies (*I*^2^ = 97.9%, *P* < 0.001); consequently, a random effects model was applied. Overall, ADL performance in the MI combined with NIBS group improved significantly compared to the control group (WMD = 8.94; 95% CI 4.36, 13.53; *P* < 0.001), as depicted in Figure 6.

### 3.5 Subgroup analysis

Subgroup analyses were stratified by stroke stage, MI paradigm, MI duration, NIBS modality (LF-rTMS, bi-tDCS, c-tDCS, or a-tDCS), stimulation duration, and control group design. The results of these analyses are summarized in Tables 3–5.

**Table 3:** Subgroup analysis of upper limb motor function.

Subgroup	Number of studies	Heterogeneity test		Meta-analysis results statistics		
		*I*^2^ (%)	*P*-value	WMD (95%CI)	*Z*-value	*P*-value
Stroke stage						
Acute	9	89.9%	<0.001	7.16 (4.18, 10.14)	4.71	<0.001
Chronic	7	89.3%	<0.001	2.95 (−1.37, 7.27)	1.34	0.180
NIBS type						
bi-tDCS	10	93.4%	<0.001	4.76 (0.97, 8.56)	2.46	0.014
a-tDCS	2	0.00%	0.974	6.52 (2.55, 10.48)	3.22	0.001
LF-rTMS	9	83.0%	<0.001	6.55 (4.32, 8.79)	5.75	<0.001
NIBS stimulation time						
≤ 20 min	8	88.9%	<0.001	3.17 (−0.51, 6.84)	1.69	<0.001
>20 min	3	93.6%	<0.001	10.13 (1.62, 18.64)	2.33	<0.001
MI parameters						
BCI-MI	5	89.7%	<0.001	1.43 (−4.12, 6.97)	0.50	0.614
General-MI	11	87.4%	<0.001	6.99 (4.49, 9.49)	5.48	<0.001
GMI	4	63.2%	0.043	7.60 (5.12, 10.09)	6.00	<0.001
MI time						
≤ 30 min	13	87.4%	<0.001	4.32 (2.10, 6.54)	3.81	<0.001
>30 min	7	85.3%	<0.001	9.12 (5.37, 12.87)	4.77	<0.001
Control group type						
MI	11	89.7%	<0.001	4.28 (1.31, 7.24)	2.83	0.005
NIBS	11	95.2%	<0.001	5.17 (1.05, 9.30)	2.46	0.014

**Table 4:** Subgroup analysis of functional activities in upper limb movement.

Subgroup	Number of studies	Heterogeneity test		Meta-analysis results statistics		
		*I*^2^ (%)	*P*-value	SMD (95% CI)	*Z*-value	*P*-value
Stroke stage						
Acute	2	0.0%	0.608	1.22 (0.82, 1.63)	5.92	<0.001
Chronic	4	78.8%	0.003	0.93 (0.18, 1.67)	2.44	0.015
NIBS type						
bi-tDCS	5	75.1%	0.003	1.02 (0.45, 1.60)	3.49	<0.001
LF-rTMS	5	72.8%	0.005	1.34 (0.76, 1.91)	4.55	<0.001
MI parameters						
BCI-MI	2	92.5%	<0.001	0.91 (−1.24, 3.06)	0.83	0.407
General-MI	4	0.0%	0.783	1.10 (0.81, 1.39)	7.54	<0.001
GMI	4	78.5%	0.003	1.41 (0.68, 2.14)	3.78	<0.001
MI time						
≤ 30 min	8	58.6%	0.018	0.96 (0.61, 1.32)	5.30	<0.001
>30 min	2	0.0%	0.655	2.05 (1.56, 2.54)	8.24	<0.001
Control group type						
MI	5	78.9%	0.001	0.98 (0.37, 1.59)	3.14	0.002
NIBS	5	66.4%	0.018	1.38 (0.85, 1.91)	5.11	<0.001

**Table 5:** Subgroup analysis of activities of daily living.

Subgroup	Number of studies	Heterogeneity test		Meta-analysis results statistics		
		*I*^2^ (%)	*P*-value	WMD (95% CI)	*Z*-value	*P*-value
Stroke stage						
Acute	10	96.8%	< 0.001	10.73 (5.44, 16.03)	3.97	< 0.001
Chronic	3	97.8%	< 0.001	4.80 (−2.59, 12.18)	1.27	0.203
NIBS type						
bi-tDCS	7	99.1%	< 0.001	9.02 (0.71,17.33)	2.13	0.033
a-tDCS	2	0.0%	0.890	6.03 (2.60, 9.46)	3.45	0.001
LF-rTMS	10	85.2%	< 0.001	9.40 (6.55, 12.24)	6.47	< 0.001
NIBS stimulation time						
≤ 20 min	5	96.4%	< 0.001	4.97 (−0.29, 10.23)	1.85	0.064
>20 min	3	99.0%	< 0.001	10.73 (−5.30, 26.76)	1.31	0.189
MI parameters						
BCI-MI	2	98.4%	< 0.001	0.84 (−6.86, 8.55)	0.21	0.830
General-MI	11	96.4%	< 0.001	11.01 (6.07, 15.95)	4.37	< 0.001
GMI	5	67.1%	0.016	9.57 (6.29, 12.85)	5.71	< 0.001
MI time						
≤ 30 min	12	94.9%	< 0.001	7.27 (3.73, 10.81)	4.03	< 0.001
>30 min	6	95.8%	< 0.001	13.23 (7.32, 19.14)	4.39	< 0.001
Control group type						
MI	7	96.9%	< 0.001	7.82 (1.58, 14.06)	2.46	0.014
NIBS	12	97.3%	< 0.001	9.55 (4.70, 14.40)	3.86	< 0.001

#### 3.5.1 Upper limb motor function

Table 3 demonstrates that the intervention yielded a significant therapeutic effect in patients in the subacute phase (WMD = 7.16; 95% CI 4.18, 10.14; *P* < 0.001); in contrast, no significant benefit was observed in patients in the chronic phase (WMD = 2.95; 95% CI −1.37, 7.27; *P* = 0.180). Regarding NIBS modalities, bi-tDCS, LF-rTMS, and a-tDCS all elicited significant therapeutic effects (see Table 3). Stratification by stimulation duration indicated that both sessions ≤ 20 min (WMD = 3.17; 95% CI −0.51, 6.84; *P* < 0.001) and >20 min (WMD = 10.13; 95% CI 1.62, 18.64; *P* < 0.001) produced significant therapeutic benefits. Analysis of MI paradigms revealed that General-MI and GMI conferred significant efficacy (*P* < 0.001 for both), whereas BCI-MI did not yield a statistically significant outcome (WMD = 1.43; 95% CI −4.12, 6.97; *P* = 0.614). Regarding MI duration, while significant improvements were noted for both < 30 min and >30 min, durations exceeding 30 min appeared to confer greater therapeutic magnitude (WMD = 9.12 and WMD = 4.32, *P* < 0.001). Finally, the combined intervention demonstrated superior efficacy compared to either monotherapy control group: MI alone (WMD = 4.28; 95% CI 1.31, 7.24; *P* = 0.005) and NIBS alone (WMD = 5.17; 95% CI 1.05, 9.30; *P* = 0.014).

#### 3.5.2 Functional activities of upper limb movement

Table 4 indicates that the intervention yielded significant therapeutic effects in both subacute (WMD = 1.22; 95% CI 0.82, 1.63; *P* < 0.001) and chronic (WMD = 0.93; 95% CI 0.18, 1.67; *P* = 0.015) stroke phases. In terms of NIBS modalities, both bi-tDCS (WMD = 1.02; 95% CI 0.45, 1.60; *P* < 0.001) and LF-rTMS (WMD = 1.34; 95% CI 0.76, 1.91; *P* < 0.001) demonstrated significant therapeutic effects. Regarding MI paradigms, both General-MI (WMD = 1.10; 95% CI 0.81, 1.39; *P* < 0.001) and GMI (WMD = 1.41; 95% CI 0.68, 2.14; *P* < 0.001) were effective, whereas BCI-MI failed to show a significant therapeutic effect (WMD = 0.91; 95% CI −1.24, 3.06; *P* = 0.407). Stratification by MI duration revealed that sessions ≤ 30 min (WMD = 0.96; 95% CI 0.61, 1.32; *P* < 0.001) and >30 min (WMD = 2.05; 95% CI 1.56, 2.54; *P* < 0.001) both produced significant therapeutic effects. Furthermore, the combined intervention proved superior to single modality controls, including MI alone (WMD = 0.98; 95% CI 0.37, 1.59; *P* = 0.002) and NIBS alone (WMD = 1.38; 95% CI 0.85, 1.91; *P* < 0.001).

#### 3.5.3 Activities of daily life

Table 5 indicates that significant therapeutic benefits were observed in the subacute phase (WMD = 10.73; 95% CI 5.44, 16.03; *P* < 0.001), whereas no statistically significant effect was found in the chronic phase (WMD = 4.80; 95% CI −2.59, 12.18; *P* = 0.203). Regarding NIBS modalities, bi-tDCS (WMD = 9.02; 95% CI 0.71, 17.33; *P* = 0.033), LF-rTMS (WMD = 9.40; 95% CI 6.55, 12.24; *P* < 0.001), and a-tDCS (WMD = 6.03; 95% CI 2.60, 9.46; *P* = 0.001) all elicited significant therapeutic effects. Stratification by stimulation duration revealed that neither sessions ≤ 20 min (WMD = 4.97; 95% CI −0.29, 10.23; *P* = 0.064) nor those >20 min (WMD = 10.73; 95% CI −5.30, 26.76; *P* = 0.189) reached statistical significance. Analysis of MI paradigms demonstrated significant efficacy for General-MI (*P* < 0.001) and GMI (*P* < 0.001). In contrast, BCI-MI did not yield a significant therapeutic effect (WMD = 0.84; 95% CI −6.86, 8.55; *P* = 0.830). Regarding MI duration, significant improvements were observed for both sessions < 30 min (WMD = 7.27; 95% CI 3.73, 10.81; *P* < 0.001) and >30 min (WMD = 13.23; 95% CI 7.32, 19.14; *P* < 0.001). Finally, the combined intervention demonstrated superior efficacy compared to either monotherapy control group, including MI alone (WMD = 7.82; 95% CI 1.58, 14.06; *P* = 0.014) and NIBS alone (WMD = 9.55; 95% CI 4.70, 14.40; *P* < 0.001).

### 3.6 Safety outcomes and adverse events

A structured synthesis of safety outcomes was conducted based on the reported adverse events (Table 2). Among the 16 included studies, 12 explicitly reported no adverse events, and 2 did not provide safety data. Adverse events were predominantly modality-specific, tDCS combined interventions: side effects were mild and transient, limited to localized tingling and itching under the electrodes. rTMS combined interventions, While generally safe, one study reported more systemic and severe adverse events, including headaches, muscle aches, negative emotions, and epileptic seizures.

Overall, the combined intervention of NIBS and MI demonstrates a highly acceptable safety profile. However, the isolated report of epileptic seizures underscores the absolute necessity of rigorous patient screening and strict adherence to safety guidelines, particularly when applying rTMS protocols.

### 3.7 Results of sensitivity analysis

**Figure 7:**
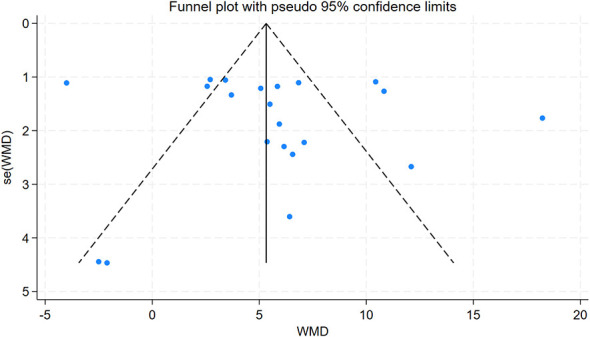
Funnel plot of upper limb motor function.

**Figure 8:**
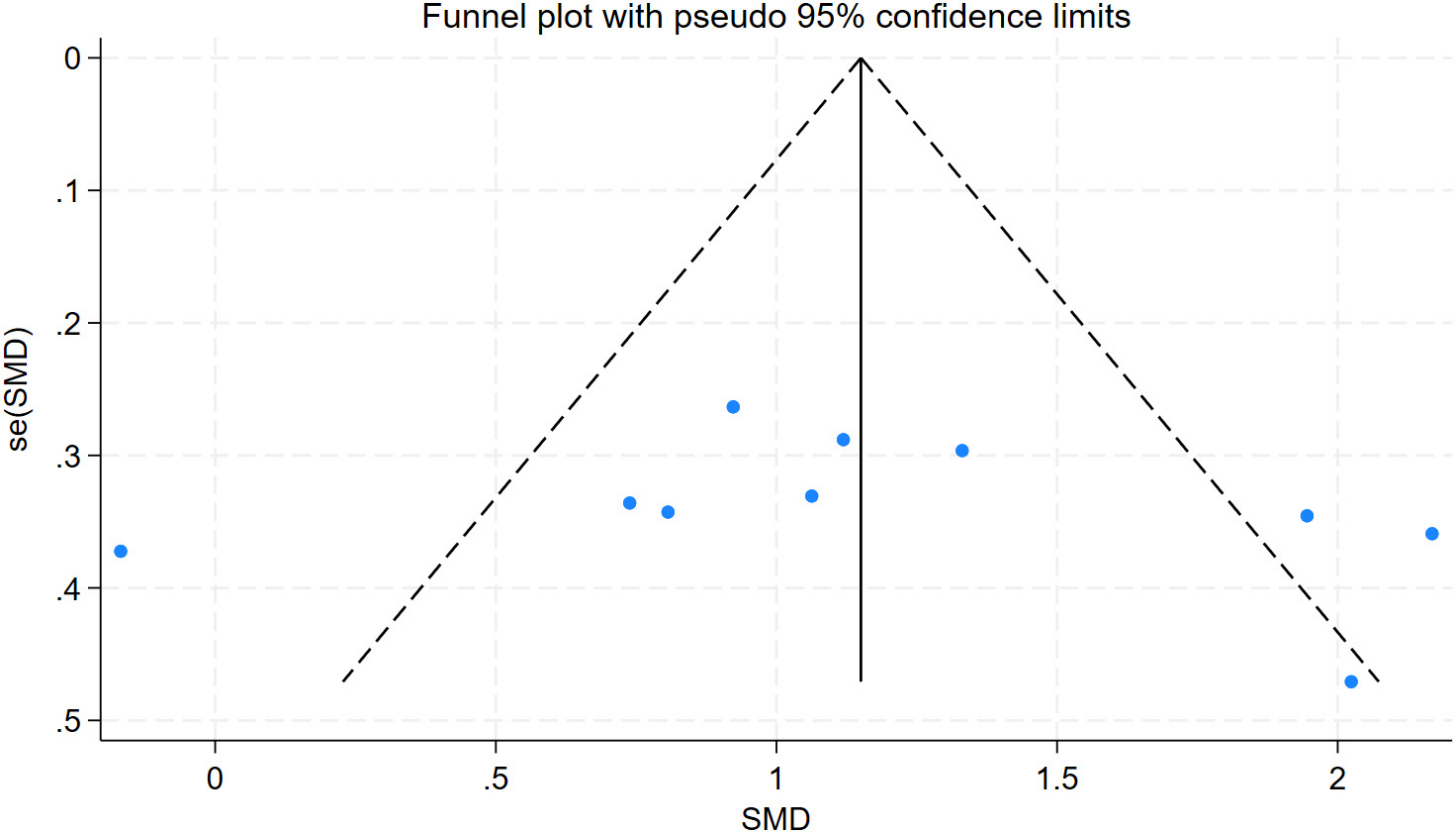
Funnel diagram of functional activities of upper limb movement.

**Figure 9:**
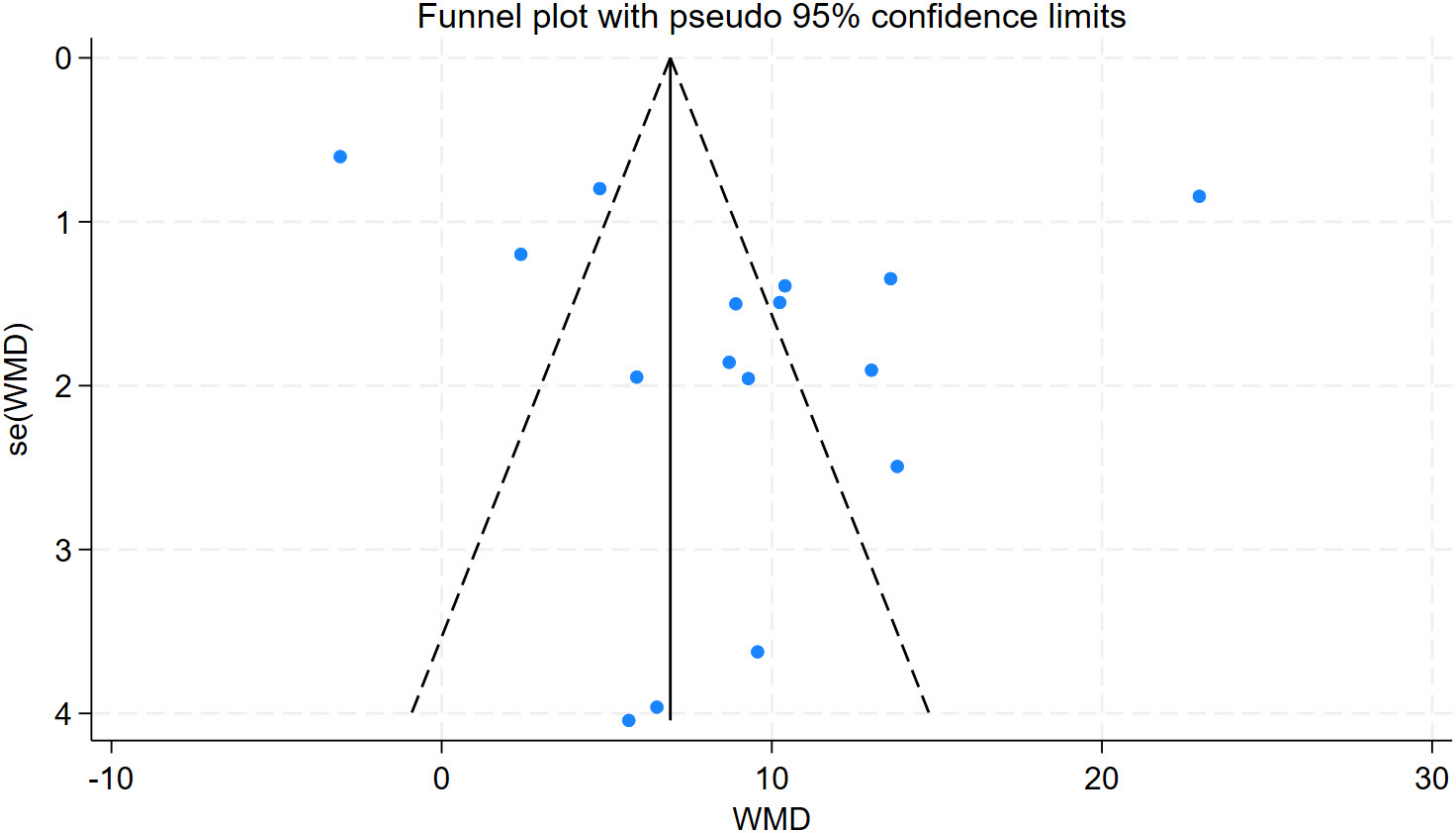
Funnel plot of activities of daily living.

Sensitivity analysis confirmed the robustness of the meta-analysis results, indicating that the pooled effect sizes were stable and not driven by any single study. Visual inspection of the funnel plots revealed a symmetrical distribution of effect sizes, suggesting a low risk of publication bias. Consistent with this, Egger's regression tests yielded *P*-values > 0.05, providing no statistical evidence of significant publication bias among the included studies. The funnel plots are presented in Figures 7–9.

## 4 Discussion

### 4.1 Results of meta-analysis

This systematic review and meta-analysis evaluated the efficacy of NIBS combined with MI for upper limb motor recovery in stroke patients, concurrently assessing therapeutic impacts on motor function, activities of daily living (ADL), and functional capacity. Subgroup analyses identified stroke chronicity, MI paradigm, MI duration, NIBS modality, intervention dosage, and comparator type as critical determinants of treatment outcomes.

Relative to previous meta-analyses (20), this study included 1,249 participants, thereby enhancing statistical power and the reliability of effect estimates. The inclusion of recent high-quality RCTs has led to a meaningful recalibration of the pooled effect size. Subgroup analysis revealed that MI duration and NIBS stimulation duration served as significant modulators of upper limb motor recovery. Specifically, a dose-response relationship was observed in FMA-UE outcomes: the WMD for MI duration >30 min (WMD = 9.69; 95% CI 8.43, 10.96) was substantially higher than that for duration ≤ 30 min (WMD = 2.50; 95% CI 1.77, 3.23). These findings diverge from prior conclusions, underscoring the necessity of continuously updating clinical guidelines to reflect the evolving evidentiary landscape.

The FMA-UE is a widely established instrument for evaluating upper limb motor impairment post-stroke (21). Analysis of 21 studies utilizing FMA-UE demonstrated that the combined NIBS and MI intervention yielded significantly superior efficacy compared to monotherapy, with effect sizes exceeding prior evidence (20). Notably, the pooled effect size for FMA-UE in the combined group (WMD = 5.75) effectively surpasses the established Minimal Clinically Important Difference (MCID) threshold of 3.5–5.25 points for stroke patients (22). This indicates that the intervention offers not only statistical significance but also clinical relevance. Clinically, this may translate to the restoration of active initiation in the shoulder joint or the refinement of distal motor control, progressing from gross grasp to precision grip. Furthermore, the combined intervention yielded significant improvements in upper limb functional capacity, as evaluated by the WMFT and ARAT. Regarding specific modalities (23), noted that while rTMS confers moderate improvements in upper limb function, the efficacy of tDCS remains relatively limited, with effects being more pronounced in the acute phase. Given that NIBS monotherapy often yields modest results, combining it with other rehabilitation strategies is recommended to potentiate therapeutic outcomes, though further high-quality RCTs are required to validate these findings.

Studies have shown that although isolated neuromodulation techniques can modulate cortical excitability and induce transient neuroplasticity, they often fail to translate into meaningful improvements in complex activities of daily living (ADL). For instance, some studies have reported that while tDCS can improve Fugl-Meyer scores, the Barthel Index remains unchanged, suggesting that mere modulation of cortical excitability is insufficient to generalize to tasks demanding motor planning and environmental interaction (15). In contrast, the synergistic application of non-invasive brain stimulation (NIBS) and motor imagery (MI) can recruit neural networks congruent with physical execution, enhancing task specificity and plasticity, thereby facilitating the restoration of ADL capacity in stroke patients. Notably, analysis revealed that MBI scores in the combined intervention group exceeded the Minimum Clinically Important Difference (MCID), indicating a significant enhancement in patients' independence in complex daily activities. This improvement represents a shift from high dependency to partial self-care, effectively enhancing quality of life and reducing the burden of care. Collectively, these findings support the adoption of a multimodal strategy in stroke rehabilitation, specifically leveraging MI as a “cognitive scaffold” to consolidate the neural excitability primed by NIBS, thereby fostering more ecologically valid functional recovery. Future research should further clarify the optimal parameters of such combined regimens to maximize recovery outcomes across different stroke populations.

### 4.2 Subgroup analysis

The high overall statistical heterogeneity (*I*^2^ > 85%) accurately mirrors the clinical and methodological diversity inherent in stroke neurorehabilitation. Instead of relying solely on the pooled global estimates, this study utilized comprehensive subgroup analyses to rigorously deconstruct this variance. Importantly, our exploration of patient characteristics revealed that the therapeutic outcomes were not significantly moderated by participant age. The vast differences in efficacy observed across trials appear to be fundamentally attributable to the intrinsic nature of the intervention modalities (e.g., stimulation parameters and MI paradigms) rather than the demographic age of the subjects.

#### 4.2.1 Stroke stage

To enhance evidence reliability, the subgroup analysis aggregated patients in the acute and subacute phases, comparing this cohort against those in the chronic phase. This stratification strategy was designed to mitigate small sample bias while maximizing statistical power. Although clinical priorities shift from neuroprotection in the acute stage to functional reconstruction in the subacute stage, there is substantial overlap in the neurobiological dynamics of brain recovery between the two (24), as both phases are characterized by relatively high neural plasticity. Consequently, contrasting this early-recovery cohort with the chronic phase elucidates the temporal dependency of the intervention effect. The results indicate that the therapeutic efficacy of NIBS combined with MI is chronicity-dependent. In the acute/subacute phase, combined treatment demonstrated significant superiority over monotherapy regarding FMA-UE and MBI outcomes, though gains in upper limb functional capacity remained limited. Conversely, in the chronic stage, combination therapy failed to yield statistically significant advantages. These findings align with the “time window” theory of neural plasticity (25). During the early post-stroke stages, the brain exhibits heightened neuroplasticity and sensitivity to NIBS and MI, thereby facilitating functional reorganization. In contrast, the chronic stage is characterized by diminished plasticity and established maladaptive compensation patterns, which may attenuate the intervention effect.

#### 4.2.2 NIBS type

Current evidence indicates that diverse NIBS modalities can effectively enhance upper limb motor function post-stroke. Consistent with the present results, the meta-analysis (20) reported that MI combined with tDCS or rTMS significantly improved upper limb motor function, functional capacity, and activities of daily living. Specifically, subgroup analyses revealed that both bi-tDCS and LF-rTMS exhibited significant and robust effects on upper limb motor recovery. However, regarding activities of daily living (ADL), LF-rTMS and bi-tDCS yielded the most substantial effect sizes, followed by a-tDCS.

This divergence in efficacy may be elucidated by the interhemispheric competition model. Conventional unilateral a-tDCS typically applies anodal stimulation exclusively to the ipsilesional hemisphere. While this effectively upregulates ipsilesional excitability, it may fail to adequately suppress the pathological hyperexcitability of the contralesional hemisphere. Conversely, LF-rTMS modulates this imbalance by selectively inhibiting the contralesional primary motor cortex (M1) via long-term depression (LTD)-like mechanisms. Post-stroke, the contralesional hemisphere frequently exhibits maladaptive hyperactivation, exerting excessive transcallosal inhibition (TCI) onto the affected hemisphere, thereby suppressing perilesional plasticity and impeding functional recovery. Consequently, modalities specifically targeting this interhemispheric imbalance—such as LF-rTMS and bi-tDCS—appear clinically superior to unilateral a-tDCS for improving ADL outcomes.

#### 4.2.3 Time factor

While prior meta-analyses (20) suggested comparable efficacy across varying durations of NIBS and MI training, the present analysis identifies intervention dosage as a critical determinant of outcomes. Specifically, NIBS sessions exceeding 20 min yielded significantly greater improvements in FMA-UE compared to shorter durations ( ≤ 20 min). Similarly, motor imagery sessions >30 min demonstrated superior efficacy in FMA-UE and MBI outcomes relative to sessions ≤ 30 min. These findings underscore a clear temporal dose-response relationship. Extended stimulation durations likely induce more robust and sustained neuroplastic changes. Neurophysiologically, the duration of tDCS aftereffects correlates positively with stimulation duration (26). Regarding MI, extended sessions are necessary to accommodate the high cognitive load required for transitioning from simple motor ideation to complex, function-oriented motor sequencing (27), a process crucial for functional gains in FMA-UE and MBI. Sessions exceeding 30 min facilitate deeper motor coding and the activation of memory networks essential for skill consolidation (28). Thus, sufficient intervention duration serves as the requisite foundation for inducing persistent neural plasticity and regulating cortical excitability. While our subgroup analyses indicate a potential trend favoring Graded Motor Imagery (GMI) and MI durations exceeding 30 min, these findings are observational and derived from indirect comparisons. They must be interpreted with extreme caution due to the small number of included studies within these specific strata (e.g., only 4 studies for GMI) and the persistently high residual heterogeneity. These results highlight promising clinical avenues rather than establishing definitive superiority.

#### 4.2.4 MI parameters

Therapeutic efficacy varied significantly across distinct MI paradigms. Both General-MI and GMI demonstrated significant clinical benefits. Notably, GMI yielded superior outcomes in motor impairment (FMA-UE), whereas General-MI proved more effective for ADL (MBI). In contrast, BCI-MI failed to demonstrate statistically significant advantages for either outcome measure. This lack of efficacy may be attributed to technical limitations; Ramos-Murguialday noted that BCI classification accuracy frequently falls below 80%, leading to erroneous feedback that may disrupt Hebbian learning and interfere with adaptive neuroplasticity (29). Therefore, optimizing Event-Related Desynchronization classification algorithms is critical to improving the clinical utility of BCI-MI.

#### 4.2.5 Control group type

Subgroup analyses confirmed that the combination of NIBS and MI conferred significant additive functional benefits compared to either monotherapy alone, with functional gains being notably superior to those achieved by NIBS monotherapy. This suggests a synergistic relationship, indicating that the adjunctive application of MI to NIBS protocols can potentiate therapeutic outcomes. Consequently, combined intervention warrants prioritization as a recommended treatment strategy where clinical feasibility permits.

### 4.3 Therapeutic mechanism

#### 4.3.1 Time window theory of neuroplasticity

Neuroplasticity encompasses the central nervous system's capacity for structural and functional adaptation, characterized by synaptic modulation and large-scale network reorganization. Central to stroke recovery, the “time window theory” posits that neuroplastic potential is dynamic rather than static. Specifically, a “critical period” of heightened sensitivity exists post-injury, during which the brain exhibits a potentiated response to therapeutic interventions (6). The acute phase is characterized by concurrent inflammatory cascades and excitotoxicity, alongside the robust activation of endogenous repair mechanisms. Early interventions such as NIBS or MI may amplify these spontaneous recovery processes, thereby driving functional reorganization within perilesional territories (30). During the subacute phase, neuroplasticity remains elevated as the brain attempts to restore connectivity via axonal sprouting and synaptic remodeling (6), making this an optimal window for functional gains. In contrast, the chronic phase (>6 months) is typically characterized by a decline in plasticity and the stabilization of lesion pathology. Consequently, therapeutic responsiveness diminishes, often necessitating higher-intensity stimulation to re-engage dormant plasticity mechanisms (24).

#### 4.3.2 Hebbian learning principle

The core tenet of Hebbian theory posits that “neurons that fire together, wire together” (31). In the context of combined NIBS and MI, NIBS primes the lesioned motor network by inducing subthreshold membrane depolarization and modulating interhemispheric inhibition, thereby establishing a state of postsynaptic facilitation. Concurrently, MI provides synchronous, task-specific presynaptic input within this primed environment. This precise temporal coordination fulfills the Hebbian criterion for coincident activation (32), thereby eliciting more robust LTP-like plasticity compared to monotherapy (19). This synergistic interaction fosters synaptic strengthening and cortical/subcortical network remodeling, effectively facilitating the consolidation of transient plasticity into sustained functional gains (33), ultimately culminating in long-lasting, clinically meaningful motor recovery.

#### 4.3.3 Time effect mechanism

The combination of NIBS and MI exhibits a significant dose-response relationship regarding upper limb motor recovery. Extending NIBS stimulation duration can induce more sustained changes in cortical excitability (34), enhance long-term potentiation (LTP)- and long-term depression (LTD)-like synaptic plasticity, and significantly prolong the “neural plasticity time window,” thereby establishing an optimal neurophysiological milieu for concurrent MI. Concurrently, MI sessions exceeding 30 min ensure sufficient capacity for high-quality kinesthetic rehearsal, facilitating the robust recruitment of motor execution networks. Together, these mechanisms foster a synergistic interaction, producing a non-linear enhancement effect on functional recovery (35).

### 4.4 Limitations

This study is subject to several limitations. First, the restriction of inclusion criteria to specific validated outcome measures (FMA-UE, MBI, WMFT, ARAT) may limit the generalizability of findings to other domains of recovery. Second, the exclusion of unpublished or gray literature introduces a potential risk of publication bias. While funnel plot asymmetry and Egger's tests did not indicate significant bias, the possibility of unreported negative findings cannot be entirely precluded, which may potentially inflate effect estimates. Third, despite sensitivity analyses confirming robustness, the persistence of high heterogeneity suggests the presence of residual clinical or methodological diversity that remains unaccounted for. Finally, the limited sample sizes within certain subgroup stratifications may constrain statistical power, warranting caution in interpreting these specific interactions. Importantly, the Risk of Bias directly impacts our certainty of evidence. As illustrated in our assessment, the lack of participant blinding is a pervasive challenge in cognitive-motor rehabilitation like MI, as the intervention inherently requires the patient's conscious mental participation. This inescapable pragmatic constraint introduces potential performance bias. Consequently, while our confidence in objective outcomes (e.g., FMA-UE) remains robust due to large effect sizes, the certainty of evidence for subjective, patient-reported metrics (such as ADL scores) is inherently downgraded, necessitating more cautious clinical interpretation. While current evidence strongly supports the clinical utility of this multimodal intervention, future direct head-to-head randomized controlled trials are urgently needed to definitively confirm the relative superiority of specific MI paradigms and stimulation dosages, thereby refining standardized clinical protocols.

## 5 Conclusion

In conclusion, this meta-analysis demonstrates that the combinatorial application of non-invasive brain stimulation and motor imagery yields significant improvements in upper limb motor function and activities of daily living in stroke patients. Crucially, subgroup analyses identified stroke chronicity, NIBS modality, stimulation dosage, MI paradigm, and comparator design as critical determinants of therapeutic efficacy. While current evidence strongly supports the clinical utility of this multimodal intervention, future research should prioritize high-quality, large-sample randomized controlled trials to further refine treatment protocols and validate long-term outcomes.

## Data Availability

The original contributions presented in the study are included in the article/Supplementary material, further inquiries can be directed to the corresponding author.
