# Supplementary material for: The effect of non-invasive brain stimulation combined with motor imagery on upper limb motor function and activities of daily living in stroke patients: a systematic review and meta-analysis
Source: Front Neurol. 2026 Jun 3;17:1807447. doi: 10.3389/fneur.2026.1807447 (PMC13271936; doi:10.3389/fneur.2026.1807447)
Supplement: Supplementary file 1 [file Supplementary_file_1.docx]

Supplementary Material

**Supplementary “Search strategy”**

(((((((((((((((((((((((((((((tDCS) OR (Anodal Stimulation Transcranial Direct Current Stimulation)) OR (Anodal Stimulation tDCS)) OR (Anodal Stimulation tDCSs)) OR (Stimulation tDCS, Anodal)) OR (Stimulation tDCSs, Anodal)) OR (tDCS, Anodal Stimulation)) OR (tDCSs, Anodal Stimulation)) OR (Cathodal Stimulation Transcranial Direct Current Stimulation)) OR (Cathodal Stimulation tDCS)) OR (Cathodal Stimulation tDCSs)) OR (Stimulation tDCS, Cathodal)) OR (Stimulation tDCSs, Cathodal)) OR (tDCS, Cathodal Stimulation)) OR (tDCSs, Cathodal Stimulation)) OR (Transcranial Alternating Current Stimulation)) OR (Transcranial Random Noise Stimulation)) OR (Repetitive Transcranial Electrical Stimulation)) OR (Transcranial Electrical Stimulation)) OR (Electrical Stimulations, Transcranial)) OR (Electrical Stimulation, Transcranial)) OR (Stimulations, Transcranial Electrical)) OR (Stimulation, Transcranial Electrical)) OR (Transcranial Electrical Stimulations)) OR ("Transcranial Direct Current Stimulation"[Mesh])) OR (((((((((Magnetic Stimulations, Transcranial) OR (Magnetic Stimulation, Transcranial)) OR (Stimulations, Transcranial Magnetic)) OR (Stimulation, Transcranial Magnetic)) OR (Transcranial Magnetic Stimulations)) OR (Transcranial Magnetic Stimulation, Paired Pulse)) OR (Transcranial Magnetic Stimulation, Repetitive)) OR (Transcranial Magnetic Stimulation, Single Pulse)) OR ("Transcranial Magnetic Stimulation"[Mesh]))) OR (rTMS)) OR (Non-Invasive Brain Stimulation)) AND ((Motor Imagery) OR (MI))) AND (((((((((((((((((((((((((((((Strokes) OR (Cerebrovascular Accident)) OR (Cerebrovascular Accidents)) OR (Cerebral Stroke)) OR (Cerebral Strokes)) OR (Stroke, Cerebral)) OR (Strokes, Cerebral)) OR (Cerebrovascular Apoplexy)) OR (Apoplexy, Cerebrovascular)) OR (Vascular Accident, Brain)) OR (Brain Vascular Accident)) OR (Brain Vascular Accidents)) OR (Vascular Accidents, Brain)) OR (Cerebrovascular Stroke)) OR (Cerebrovascular Strokes)) OR (Stroke, Cerebrovascular)) OR (Strokes, Cerebrovascular)) OR (Apoplexy)) OR (CVA (Cerebrovascular Accident))) OR (CVAs (Cerebrovascular Accident))) OR (Stroke, Acute)) OR (Acute Stroke)) OR (Acute Strokes)) OR (Strokes, Acute)) OR (Cerebrovascular Accident, Acute)) OR (Acute Cerebrovascular Accident)) OR (Acute Cerebrovascular Accidents)) OR (Cerebrovascular Accidents, Acute)) OR ("Stroke"[Mesh]))
